# The Evils Arising from Tight-Lacing

**Published:** 1860-07

**Authors:** S. C. Young


### ARTICLE XIX.

*The Evils Arising from Tight-Lacing*, from the subject of an article of DR. S. C. YOUNG in the New Orleans Medical and Surgical Journal for May.

THE author considers the evils of tight-lacing as affecting—1. The osseous system. 2. The thoracic viscera. 3. The abdominal viscera. 4. The pelvic viscera.

1. The principal parts of the osseous system are the spinal column and the ribs. The former rarely becomes affected from this cause; when, however, it becomes so, the injury is of a serious nature—caries or necrosis. The ribs are more frequently injured, the most frequent result being the deformity called "chicken-breast." This means a turning inwards of the ribs from continued pressure from without, materially diminishing the space occupied by the lungs. The angle of the ribs is thereby made more acute, and they are rendered more liable to fracture.

2. Among the thoracic viscera, the *lungs* suffer most from this abominable fashion. They are not allowed to expand to their full capacity—the arterialization of the blood is interfered with: cold feet, cold hands, head aches, nervousness, debility, dullness and weakness of mind, are the result of a deficiency of oxygen in the blood. The lungs become predisposed to tubercular deposit by tight-lacing, and chronic enlargement of the air-cells, produce a state similar to what is called the "heaves" in horses.

The heart, of course, suffers at the same time; it is over-taxed, becomes first irregular, and later, and not unfrequently, the seat of organic disease.

3. The liver, stomach, and intestines, suffer among the abdominal organs. The liver is crowded in an unnatural position and shape, its circulation obstructed, the glycogenic function interfered with, and thus the foundation is laid to many acute and chronic disorders.

The stomach suffers with the other organs in the general debility; it is prevented from developing itself normally, and its rotatory and peristaltic action is interfered with. The morbid and vitiated appetite of our fashionable tight-lacing ladies is frequently attributable to this cause. The pressure upon the intestines produces various evil results. It diminishes the peristaltic motion, obstructs the circulation, especially the portal; interferes with the proper performance of the function of the absorbents, and, by diminishing the abdominal cavity, forces the intestines into the pelvis; in this way—

4. The bladder and the uterus are made to suffer. The capacity of the former is diminished, and either frequent micturation or retention of the urine may result, and predispose to the deposition of stone.

More harm is done by pressure on the uterus than on any other organ. The whole catalogue of retro and ante-versions, retro and ante-flexions, prolapsus and procidentia uteri, find in tight-lacing a predisposing as well as an exciting cause. Abortions and miscarriages, with their long train of bodily and mental suffering, are produced by pressure upon the abdomen from this miserable practice.

We have perused the paper of Dr. Young with great pleasure, and fully agree with him when he remarks, that it is the imperative duty of the physician to teach woman "how much better it is to *live* unfashionably than to *die* fashionably."

